# Adsorption of the Inflammatory Mediator High-Mobility Group Box 1 by Polymers with Different Charge and Porosity

**DOI:** 10.1155/2014/238160

**Published:** 2014-08-27

**Authors:** Carla Tripisciano, Tanja Eichhorn, Stephan Harm, Viktoria Weber

**Affiliations:** ^1^Christian Doppler Laboratory for Innovative Therapy Approaches in Sepsis, Department for Health Sciences and Biomedicine, Danube University Krems, Dr.-Karl-Dorrek-Straße 30, 3500 Krems, Austria; ^2^Center for Biomedical Technology, Department for Health Sciences and Biomedicine, Danube University Krems, Dr.-Karl-Dorrek-Straße 30, 3500 Krems, Austria

## Abstract

High-mobility group box 1 protein (HMGB1) is a conserved protein with a variety of biological functions inside as well as outside the cell. When released by activated immune cells, it acts as a proinflammatory cytokine. Its delayed release has sparked the interest in HMGB1 as a potential therapeutic target. Here, we studied the adsorption of HMGB1 to anionic methacrylate-based polymers as well as to neutral polystyrene-divinylbenzene copolymers. Both groups of adsorbents exhibited efficient binding of recombinant HMGB1 and of HMGB1 derived from lipopolysaccharide-stimulated peripheral blood mononuclear cells. The adsorption characteristics depended on particle size, porosity, accessibility of the pores, and charge of the polymers. In addition to these physicochemical parameters of the adsorbents, modifications of the molecule itself (e.g., acetylation, phosphorylation, and oxidation), interaction with other plasma proteins or anticoagulants (e.g., heparin), or association with extracellular microvesicles may influence the binding of HMGB1 to adsorbents and lead to preferential depletion of HMGB1 subsets with different biological activity.

## 1. Introduction

High-mobility group box 1 protein (HMGB1) is a ubiquitous nonhistone DNA binding protein with distinct intra- and extracellular functions. It is crucial for nuclear architecture and has been implicated in DNA replication, repair, and transcription. It acts as a sentinel for nucleic acid-mediated immune responses [1, 2] and as a pathogenic inflammatory mediator during sterile and infectious injury [3–6].

Extracellular HMGB1 is either derived from passive release by injured or necrotic cells or derived from active secretion by immune cells, such as monocytes and macrophages [7–9], or natural killer cells [10, 11] after exposure to pathogen-associated molecular patterns including lipopolysaccharide (LPS) and inflammasome agonists [12, 13]. Secretion of HMBG1 from monocytes/macrophages starts 8–12 h after ligation of cell surface receptors, which represents a significantly delayed release as compared to most other inflammatory mediators produced by these cells, fostering interest in HMGB1 as a target for therapy [3, 14, 15].

HMGB1 secretion is regulated by phosphorylation and acetylation of its two nuclear localization sequences (NLS) [8, 16, 17]. Cell stress and inflammation induce NLS acetylation of HMGB1, resulting in its cytoplasmic accumulation, loading into secretory lysosomes, and release by exocytosis [18]. Secreted HMGB1 acts through various pattern-recognition receptors including the receptor for advanced glycation end products (RAGE), toll-like receptors TLR-2, TLR-4, and TLR-9, T-cell immunoglobulin domain and mucin domain 3 (TIM-3), and CXC chemokine receptor type 4 (CXCR-4) [19–24].

While the secretion of HMGB1 is regulated by phosphorylation and acetylation, its extracellular biological activity and interaction with different receptors depend on the redox state of three conserved cysteine residues at positions 23, 45, and 106. With these residues in a reduced form, HMGB1 induces chemotaxis. With a disulfide bridge between C23 and C45 and a free sulfhydryl at position 106, HMGB1 interacts with toll-like receptor 4 (TLR-4) to stimulate cytokine production, while it loses its biological activity in its completely oxidized form [25, 26].

The depletion of HMGB1 by extracorporeal therapies, such as hemofiltration with porous membranes [27] or hemoperfusion with adsorption columns has been reported [28, 29]. As HMGB1 possesses two DNA binding domains that interact with negatively charged groups, we tested different anionic polymers for their ability and capacity to bind HMGB1, compared their adsorption efficiency to neutral polystyrene divinylbenzene-based polymers, and correlated the binding characteristics to the physicochemical properties of the polymers. We show here that porosity, size distribution, hydrophobicity, and effective charge density as well as the distribution and accessibility of functional groups on the adsorbent surface are critical determinants of the adsorption characteristics. This implies that a given polymer may preferentially bind subsets of molecules with different posttranslational or oxidative modifications and with different biological activity.

## 2. Materials and Methods

### 2.1. Chemicals and Reagents

Recombinant human HMGB1 was purchased from R&D Systems (Minneapolis, USA). Cell culture medium 199 (M199), phosphate-buffered saline (PBS), 4-(2-hydroxyethyl)-1-piperazineethanesulfonic acid (HEPES), penicillin-streptomycin (PS), and lipopolysaccharide (LPS) from* E. coli* (O55:B5) were purchased from Sigma-Aldrich (St. Louis, MO, USA). Unfractionated heparin (5000 IU/mL) was from Baxter (Vienna, Austria).

### 2.2. Plasma

Venous blood was drawn into tubes containing 3.8% trisodium citrate (Vacuette, Greiner Bio-One, Kremsmuenster, Austria) from healthy adult volunteers after written informed consent. Plasma was obtained by centrifugation of the whole blood at 2000 ×g for 10 min at room temperature.

### 2.3. Adsorbents

Negatively charged and neutral adsorbents of different particle sizes and porosities were tested in this study. PSDVB-16 and PSDVB-30 (trade names CG161 and CG300; both from Rohm & Haas/Dow Chemical), as well as PVP-PSDVB (trade name Cytosorb; Cytosorbents Corporation), are hydrophobic neutral resins that are under evaluation or already in clinical application as selective cytokine adsorbents in extracorporeal blood purification. DALI (Fresenius Medical Care, Bad Homburg, Germany) and ReliSorb (Resindion S.r.l.) are both methacrylate-based polymers functionalized with polyacrylate. DALI is clinically applied for whole blood lipid apheresis. As a third negatively charged polymer, we used the cellulose-based adsorbent Cellufine sulfate (Chisso Corporation), while the neutral Cellufine GCL-2000 served as negative control. Prior to the adsorption studies, adsorbents were extensively washed with pyrogen-free 0.9% NaCl and stored at 4°C in saline solution until further use.

### 2.4. Adsorbent Characterization

Particle morphology was analyzed by scanning electron microscopy (SEM) using a TM-1000 Tabletop Microscope (Hitachi, Tokyo, Japan). Samples were washed with 100 vol% ethanol and dried for 12 h at 100°C in a heating cabinet. Adsorbents were subsequently sputter-coated with gold (Q150R ES, Quorum Technologies). Cellulose-based adsorbents were incubated overnight with 2.5 vol% glutaraldehyde, rinsed with dH_2_O, and dehydrated with increasing concentrations of ethanol (30 to 100 vol%) before sputter coating.

To determine the specific surface and the pore size distribution, nitrogen adsorption and desorption isotherms were recorded at −196°C and at relative pressures *P*/*P*_0_ between 0.001 and 1.0 using an ASAP 2010 V2.00 C physisorption analyzer (Micrometrics Instrument Corp., Norcross, USA). The Brunauer-Emmett-Teller (BET) equation [30] was used to calculate the specific surface area (*S*_BET_). The micropore volume (pore size < 2 nm) was calculated with the Horvath-Kawazoe (H-K) method [31], whereas the mesopore and macropore volume (2–50 and 50–300 nm, resp.) was obtained via the Barrett-Joyner-Halenda method (BJH) [32]. Assuming cylindrical pore geometry, the average pore diameter *d* was calculated as *d* = 4*V*/*S*_BET_ (with *V* = maximum adsorbed nitrogen volume). Values for the average particle diameter and the charge density of the negatively charged adsorbents were provided by the manufacturers.

### 2.5. Adsorption of Recombinant HMGB1

Adsorption of HMGB1 to the different polymers was studied in batch experiments using adsorbent-to-plasma ratios of 1, 5, and 10 vol%. Plasma was spiked with recombinant human HMGB1 to a target concentration of 200 ng/mL and incubated with the adsorbents at 37°C with gentle shaking. Spiked plasma without adsorbent served as a control. Samples were taken after 15 and 60 min and centrifuged immediately at 4600 ×g for 5 min to remove the adsorbents. Supernatants were collected, aliquoted, and stored at −80°C until further analysis. All experiments were conducted in triplicate. HMGB1 was quantified by enzyme-linked immunosorbent assay (ELISA, Shino-Test Corporation, Kanagawa, Japan) according to instructions of the manufacturer.

### 2.6. Adsorption of HMGB1 Derived from Stimulation of Peripheral Blood Mononuclear Cells

Human peripheral blood mononuclear cells (PBMCs) were isolated from whole blood anticoagulated with heparin (5 IU/mL final concentration) by density gradient centrifugation (Ficoll-Paque PLUS, GE Healthcare, Uppsala, Sweden) as described [33]. Cells were suspended in medium M199 supplemented with 10 vol% human plasma, 0.02 M HEPES, and 100 *μ*M PS. Aliquots of 1 × 10^6^ cells per mL of medium were stimulated with LPS (0.01–1000 ng/mL) for 16 h in HydroCell Surface 24-well plates (1 mL/well) in humidified atmosphere (5 vol% CO_2_, 37°C). After stimulation, the cells were pelleted by centrifugation, the supernatant (conditioned medium) was harvested, and the concentration of released HMGB1 was quantified by ELISA. Conditioned medium was incubated for 60 min with Cellufine sulfate or PSDVB-16, respectively, at an adsorbent-to-plasma ratio of 5 vol%. Adsorbents were removed by centrifugation and HMGB1 remaining in the supernatant was quantified by ELISA.

## 3. Results and Discussion

### 3.1. Physicochemical Characterization of the Adsorbents

The characteristics of the adsorbents used in this study are summarized in Table 1. The neutral polymers PSDVB-16 and PSDVB-30 are mesoporous polystyrene-divinylbenzene copolymers with average pore sizes of 15 and 30 nm, respectively. The smaller mean pore size of PSDVB-16 is reflected by its higher specific surface area in comparison to PSDVB-30. PVP-PSDVB, which is based on polystyrene-divinylbenzene coated with polyvinylpyrrolidone, exhibited the lowest pore diameter of all hydrophobic resins tested.

The two anionic methacrylate-based adsorbents DALI and ReliSorb differ not only with respect to their surface morphology [34], but also with respect to charge density, which is 530 and 300 *μ*equivalents of COOH per mL of dry adsorbent for DALI and ReliSorb, respectively. Cellufine sulfate, the third anionic polymer used in this study, has an approximately tenfold higher charge density.

**Figure 1:**
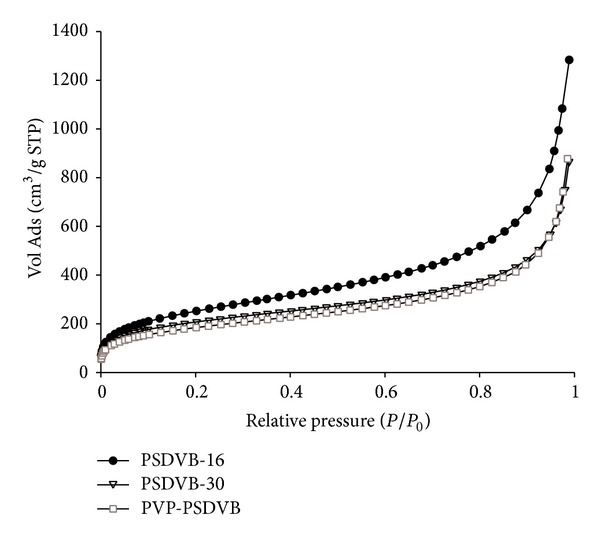
Nitrogen adsorption isotherms of uncharged polymers. Vol Ads: volume of nitrogen adsorbed; STP: standard temperature and pressure.

Adsorption isotherms were obtained by measuring the amount of N_2_ adsorbed across a wide range of relative pressures at a constant temperature. The isotherms resembled type IV according to the classification by Brunauer et al. [35], typically occurring on porous adsorbents with pores in the range of 1.5–100 nm. At lower pressures, the slope of the isotherms is given by micropores, while at higher pressures the slope reflects an increased uptake of adsorbate as pores become filled, with the inflection point typically occurring near the completion of the first monolayer (Figure 1). The neutral polystyrene-divinylbenzene-based adsorbents showed a high *S*_BET_ resulting from the presence of micro-, meso-, and macropores.

**Figure 2:**
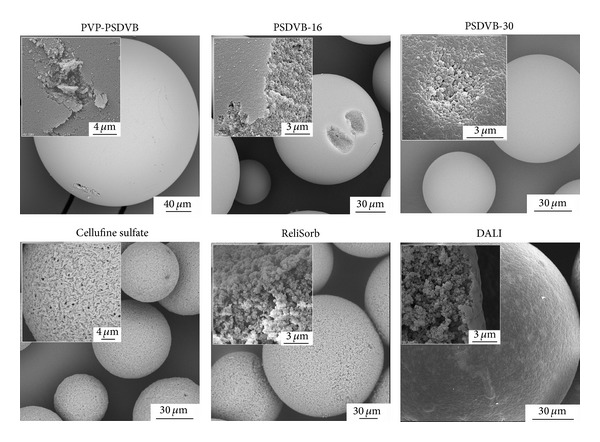
Electron micrographs of adsorbents used in this study.

The morphology of the adsorbent particles was characterized by scanning electron microscopy (SEM; Figure 2). DALI and ReliSorb, the two methacrylate-based polymers, exhibited a comparably structured inner surface but showed clear differences with respect to their outer surface, which appeared open and porous on scanning electron micrographs for ReliSorb, while it had a closed and smooth appearance for DALI. The polystyrene-divinylbenzene-based resins all had a smooth outer surface but differed with respect to their porosity, which was highest for PSDVB-30 in accordance with nitrogen adsorption measurements.

### 3.2. Adsorption of Recombinant HMGB1

**Figure 3:**
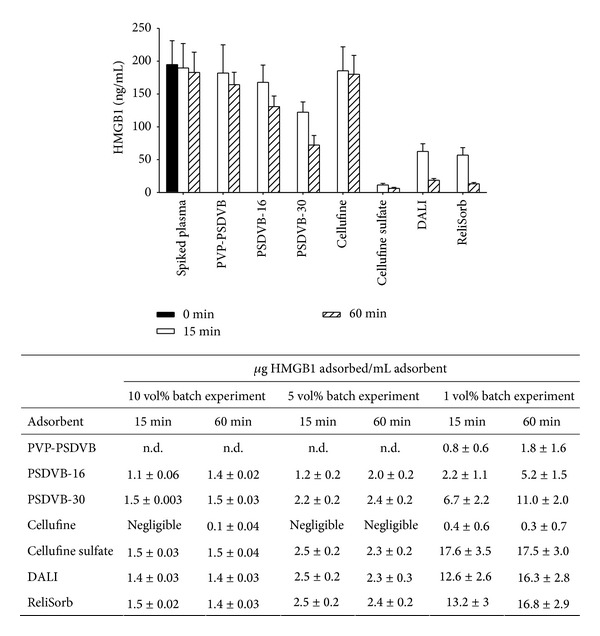
Adsorption of recombinant HMGB1. The graph shows the amount of HMGB1 remaining in spiked plasma after 15 and 60 min of incubation at an adsorbent-to-plasma ratio of 1 vol%. The amount of HMGB1 adsorbed to the polymers at adsorbent-to-plasma ratio of 1, 5, and 10 vol% is summarized in the table. Results are expressed as mean values ± standard deviation of three experiments.

HMGB1 adsorption was studied both with recombinant human HMGB1 and with HMGB1 derived from stimulated peripheral blood mononuclear cells. Pathological blood levels of HMGB1 have been reported to range between 10 and 150 ng/mL [36]. Therefore, we used a target concentration of 200 ng/mL of recombinant HMGB1 in the adsorption experiments, which are summarized in Figure 3. HMGB1 was efficiently adsorbed by both neutral and anionic polymers. Binding of HMGB1 to neutral PSDVB beads occurs via hydrophobic interactions between the amphipathic adsorbate and the hydrophobic matrix, as confirmed by the negligible HMGB1 adsorption by neutral hydrophilic cellulose beads. Regarding the polystyrene-divinylbenzene-based polymers, HMGB1 adsorption was higher for PSDVB-30 as compared to PSDVB-16, most likely due to a better accessibility of the pores due to the higher average pore diameter. PVP-PSDVB bound significantly less HMGB1 than the uncoated PSDVB polymers under identical experimental conditions despite an equivalent specific surface area. This lower efficacy can be attributed to a smaller average pore diameter as well as to diminished accessibility of the inner surface due to the coating of the polymer with polyvinylpyrrolidone. Adsorption of albumin, the most abundant plasma protein, was evaluated for the 5 vol% batch experiment. Plasma albumin levels were reduced by 1.4% to 3% for the negatively charged hydrophilic polymers and by 8% to 10% for neutral hydrophobic resins (Table 2).

The adsorption of cytokines of higher molecular mass, in particular tumor necrosis factor alpha (TNF-*α*; 51 kDa), to PVP-PSDVB polymers is considerably reduced due to a lack of accessibility of their inner surface [37, 38]. The same may hold true for HMGB1 (30 kDa), and as discussed further below, its association with a wide range of plasma proteins as well as with microvesicles may additionally influence its binding characteristics. PSDVB-based resins are clinically applied in extracorporeal liver support to remove albumin-bound metabolites such as unconjugated bilirubin [39–41]. Noteworthy, blocking of HMGB1 activity has been shown to improve hepatocyte regeneration after ischemia/reperfusion injury [2], suggesting that its removal by extracorporeal liver support systems may provide a benefit for liver regeneration.

As a DNA-associated protein, we reasoned that HMGB1 would bind to anionic surfaces via electrostatic interactions. Confirming this assumption, all anionic polymers showed efficient binding of HMGB1, with the highest efficiency for Cellufine sulfate, which has a high negative charge density and a small particle diameter, resulting in a large outer surface. Application of Cellufine sulfate as adsorbent for HMGB1 in rat hemoperfusion models resulted in decreased HMGB1 serum levels and improved survival in rats with ischemia-reperfusion injury [28, 29].

The two methacrylate-based adsorbents bound similar amounts of HMGB1 as Cellufine sulfate after 60 min (17.5 ± 3.0 versus 16.3 ± 2.8 versus 16.8 ± 2.9 *μ*g for Cellufine sulfate, DALI, and ReliSorb, resp.). The delayed binding of HMGB1 to DALI and ReliSorb as indicated by the higher remaining concentrations after 15 min may be due to differences in particle size, with the most favourable surface-to-volume ratio for Cellufine sulfate. This indicates rapid binding of HMGB1 to the adsorbent surface, while diffusion and adsorption to functional groups inside the particles occur gradually over time. DALI is clinically applied to remove low-density lipoproteins in patients with familial hypercholesterolemia, and the adsorption of HMBG1 may provide an additional benefit in the setting of atherosclerosis, which has been shown to trigger the release of HMGB1 from macrophages [42].

### 3.3. Adsorption of HMGB1 Derived from Stimulated Peripheral Blood Mononuclear Cells

**Figure 4:**
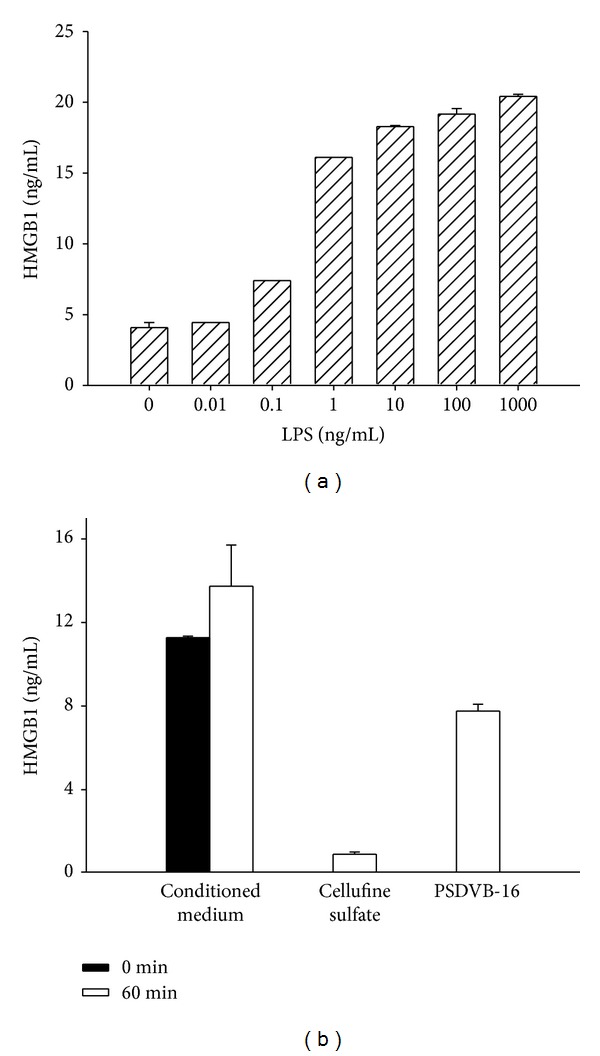
(a) Release of HMGB1 after stimulation of PBMCs with increasing concentrations of lipopolysaccharide from* E. coli* for 16 h; (b) PBMC-derived HMGB1 remaining in conditioned medium after 60 min of incubation at an adsorbent-to-medium ratio of 5 vol%; *n* = 3.

Stimulation of PBMCs with LPS resulted in a time-dependent release of HMGB1 reaching a peak after 16 h. PBMCs treated with increasing LPS concentrations (0.01–1000 ng/mL) for 16 h released HMGB1 in a concentration dependent manner (Figure 4(a)). PBMC-derived HMGB1 was efficiently removed by both adsorbents tested (243 versus 113 ng/mL adsorbent for Cellufine sulfate and PSDVB-16, respectively, for an initial concentration of 13.7 ng/mL and an adsorbent-to-medium ratio of 5 vol%, Figure 4(b)).

Next to the physicochemical parameters of the adsorbent polymers, modifications of HMGB1 are likely to influence its binding to different polymers. Acetylation, phosphorylation, and oxidation are critical for the diverse biological activities of HMGB1. In an inflammatory environment, the production of reactive oxygen species induces HMGB1 oxidation, leading to a loss of its biological activity and restricting its proinflammatory role in a temporal and spatial manner. Since acetylation, phosphorylation, and oxidation induce changes in charge and conformation of HMGB1, they might influence its binding to different polymers. It is, thus, conceivable that adsorbents preferentially deplete certain subsets of HMGB1 with different biological activity.

Finally, HMGB1 is associated with microvesicles from both activated and apoptotic cells [43]. It may be embedded into microvesicles or interact with negatively charged phosphatidylserine residues on the microvesicle surface. In any case, this association influences the adsorption of HMGB1 to porous polymers due to increased size of the adsorbate, and different subsets of HMGB1 may preferentially be associated with microvesicles, again leading to selective depletion of HMGB1 variants.

## 4. Conclusions

Neutral and anionic polymers were tested for their capability to remove recombinant as well as PBMC-derived HMGB1 from human plasma. HMGB1 was efficiently adsorbed by negatively charged beads in a time-dependent manner due to electrostatic forces. It also bound to neutral porous polystyrene-based particles via hydrophobic interactions. Unmodified hydrophilic cellulose adsorbed only negligible amounts of the cytokine, despite its large pores and high surface-to-volume ratio, while Cellulose sulfate bound HMGB1 with high efficiency.

Posttranslational modification and/or oxidation of HMGB1 are critical for the regulation of its biological activity. Since these modifications influence the charge as well as the conformation of HMGB1, subsets with different biological activity may show preferential interaction with adsorbent polymers or with plasma proteins deposited on these polymers, which might open a way to selectively deplete HMGB1 subsets with different biological activity.

## Figures and Tables

**Table 1:** Physicochemical characteristics of the adsorbents used in this study.

Polymer	Core	Ligand	Particle size [*μ*m]	Pore size [nm]	Surface area [m^2^/g]	Total *V*_pores_ [mL/g]
PVP-PSDVB	PS-DVB coated with polyvinylpyrrolidone	None	450	0.8–5	850	1.4
PSDVB-16	Polystyrene-divinylbenzene	None	120	15	900	2.1
PSDVB-30	Polystyrene-divinylbenzene	None	50–100	30	700	1.5
Cellufine	Cross-linked cellulose	None	40–130	n.d.	n.d.	n.d.
Cellufine sulfate	Cross-linked cellulose	Sulfate ester	40–120	n.d.	n.d.	n.d.
DALI	Polymethacrylamide	Polyacrylate	150–230	~180	50	1.4
ReliSorb	Polymethacrylamide	Polyacrylate	150–230	~200	28	1.7

n.d.: not determined; PSDVB: polystyrene-divinylbenzene.

**Table 2:** Albumin adsorption from plasma after 60 min of incubation at an adsorbent-to-plasma ratio of 5 vol%; *n* = 3.

Adsorbent	Albumin adsorbed [mg/mL adsorbent]	Albumin remaining [% of initial concentration]
PSDVB-16	76.6 ± 3.2	90.0 ± 0.8
PSDVB-30	62.7 ± 7.1	91.8 ± 0.5
Cellufine	10.1 ± 11	98.7 ± 1.4
Cellufine sulfate	10.8 ± 3.2	98.6 ± 0.4
DALI	19.0 ± 3.1	97.5 ± 0.3
ReliSorb	24.7 ± 3.1	96.7 ± 0.6
